# Is hepatic lipid metabolism of beef cattle influenced by breed and dietary silage level?

**DOI:** 10.1186/1746-6148-10-65

**Published:** 2014-03-12

**Authors:** Ana Sofia Henriques da Costa, Rui José Branquinho Bessa, Virgínia Maria Rico Pires, Eva Alves Rolo, Rui Manuel Amaro Pinto, Carlos Mendes Godinho Andrade Fontes, José António Mestre Prates

**Affiliations:** 1 CIISA, Faculdade de Medicina Veterinária, Universidade de Lisboa, Av. da Universidade Técnica, Pólo Universitário do Alto da Ajuda, 1300-477 Lisboa, Portugal; 2 iMed.UL, Faculdade de Farmácia, Universidade de Lisboa, Lisboa, Portugal

**Keywords:** Liver, Beef cattle, Fatty acids, Gene expression

## Abstract

**Background:**

In ruminants, unsaturated dietary fatty acids are biohydrogenated in the rumen and are further metabolised in various tissues, including liver, which has an important role in lipid and lipoprotein metabolism. Therefore, manipulation of muscle fatty acid composition should take into account liver metabolism. In the present study, the influence of breed and diet on liver lipid composition and gene expression was investigated in order to clarify the role of this organ in the lipid metabolism of ruminants. Forty purebred young bulls from two phylogenetically distant autochthonous cattle breeds, Alentejana and Barrosã, were assigned to two different diets (low *vs*. high silage) and slaughtered at 18 months of age. Liver fatty acid composition, mRNA levels of enzymes and transcription factors involved in lipid metabolism, as well as the plasma lipid profile, were assessed.

**Results:**

In spite of similar plasma non-esterified fatty acids levels, liver triacylglycerols content was higher in Barrosã than in Alentejana bulls. Moreover, the fatty acid composition of liver was clearly distinct from the remaining tissues involved in fatty acid metabolism of ruminants, as shown by Principal Components Analysis. The hepatic tissue is particularly rich in α-linolenic acid and their products of desaturation and elongation. Results indicate that *DGAT1*, *ELOVL2*, *FADS1* and *FADS2* genes influence the fatty acid composition of the liver the most. Moreover, genes such as *DGAT1* and *ELOVL2* appear to be more sensitive to genetic background than to dietary manipulation, whereas genes encoding for desaturases, such as *FADS1*, appear to be modulated by dietary silage level.

**Conclusions:**

Our results indicate that liver plays an important role in the biosynthesis of n-3 LC-PUFA. It is also suggested that dietary silage level influences the hepatic fatty acid metabolism in a breed-dependent manner, through changes in the expression of genes encoding for enzymes associated with the desaturation and elongation pathway. The importance of devising custom-made feeding strategies taking into account the genetic background is, therefore, stressed by the results from this experiment.

## Background

Despite the predominant role of adipose tissue in ruminant’s *de novo* fatty acid synthesis, the liver also plays an important role in ruminant lipid metabolism [1]. This organ carries out central metabolic functions in various aspects of lipid and lipoprotein metabolism, such as uptake, oxidation and metabolic conversion of non-esterified fatty acids (NEFA), synthesis of cholesterol and phospholipids, and formation and secretion of specific classes of lipoproteins [1]. The ruminants’ liver removes little or no triacylglycerols from blood lipoproteins [2]. Uptake of NEFA is the predominant route by which fatty acids are supplied to the liver [3] and, thus, plasma lipid fatty acid composition should influence the liver fatty acid metabolism and composition [2]. Consequently, the regulation of these liver metabolic pathways may affect fatty acid deposition into lipids of ruminant products [4].

Interest in n-3 long-chain polyunsaturated fatty acids (n-3 LC-PUFA) has increased since it was found that their consumption in most Western populations, particularly those of eicosapentaenoic acid (EPA) and docosahexaenoic acid (DHA), is sub-optimal for protection against the most prevalent chronic diseases [5]. In grazing ruminants, α-linolenic acid content of muscles increases with the concomitant increase in n-3 LC-PUFA contents [6]. In contrast, although the addition of linseed to ruminant diets [7,8] increases the α-linolenic acid content of muscles, the n-3 LC-PUFA levels stay unchanged or increase only slightly. In fact, Bessa *et al*. [9] reported that lucerne supplementation with linseed oil promoted an increase in ALA coupled with a decrease in n-3 LC-PUFA in lambs, when compared to the control diet (lucerne only). According to the authors, these results suggest the inhibition of α-linolenic acid metabolism by vegetable oils rich in n-3 PUFA. Therefore, the abundance of n-3 LC-PUFA in ruminants’ tissues appears to depend not only on dietary n-3 PUFA but also on their endogenous synthesis via elongation and desaturation of dietary n-3 PUFA.

The biosynthesis of DHA from α-linolenic acid is performed through alternating steps of desaturation and elongation, followed by a final step of peroxisomal β-oxidation. This metabolic pathway involves two desaturases (Δ5 and Δ6 desaturases), two elongases (elongases 2 and 5) and enzymes of the peroxisomal β-oxidation [10]. The activity of these enzymes is currently regarded as potential limiting steps in this biosynthesis, possibly in a tissue dependent manner. However, recent studies addressed the role of the liver in ruminants’ lipid metabolism, either using *in vitro* experiments [4] or *in vivo* assays [11]. These experiments raised some interesting clues on hepatic lipid metabolism, namely the extensive catabolism of α-linolenic acid [4] and the low or negligible expression level of genes encoding for enzymes of fatty acid desaturation and elongation [11]. Therefore, the role of bovine liver, as a central metabolic organ, on lipid metabolism remains to be elucidated.

An experiment with 40 young bulls from two genetically diverse beef cattle breeds, Alentejana and Barrosã, fed either high (70% silage/30% concentrate) or low (30% silage/70% concentrate) silage diets was carried out by our group to study the breed and diet effects on lipid metabolism. Previous reports from this experiment [12,13] showed that these breeds have a distinct response to the variation in dietary silage level, as assessed by the fatty acid composition and the mRNA levels of key lipogenic factors of the main fat depots and muscle. Bearing this in mind, as well as the studies by Gruffat *et al*. [4] and Cherfaoui *et al*. [11], we aimed to investigate whether the same breed-specific response to dietary silage level would be observed in the liver. For this purpose, the detailed fatty acid composition of liver from Alentejana and Barrosã bulls, in parallel to their mRNA levels of key genes associated with lipid metabolism, were determined.

## Results

### Body composition and plasma metabolites

The body composition parameters and plasma metabolites are depicted in Table 1. Initial and slaughter weights were higher for Alentejana when compared to Barrosã bulls (*P* < 0.001). Liver total lipids content was higher in Barrosã than in Alentejana bulls (*P* < 0.01). Both aminotransferases, AST and ALT, were higher in Alentejana than in Barrosã bulls (*P* < 0.05). The high silage fed bulls had higher ALT plasma levels than those fed the low silage diet (*P* < 0.05). Liver weight, when expressed relatively to carcass weight, was similar across experimental groups (*P* > 0.05). All plasma lipid parameters analysed were similar, regardless of breed or diet (*P* > 0.05). The ALP plasma levels were not influenced by breed or dietary silage level (*P* > 0.05).

**Table 1:** Body composition parameters and plasma metabolites

	**Alentejana**		**Barrosã**		***P *** **value**		
	**HS**	**LS**	**HS**	**LS**	**Breed**	**Diet**	**Breed × diet**
*Growth and body composition*							
Initial weight (kg)	267 ± 16.8	264 ± 13.9	210 ± 4.5	214 ± 5.9	<0.001	0.954	0.792
Slaughter weight (kg)	622 ± 17.7	636 ± 29.7	457 ± 8.9	497 ± 23.0	<0.001	0.207	0.542
Normalized liver weight (% carcass weight)	2.08 ± 0.061	1.95 ± 0.128	2.08 ± 0.052	2.01 ± 0.063	0.701	0.226	0.662
Hepatic total lipids (mg/100 g liver)	2.99 ± 0.114	2.96 ± 0.079	3.29 ± 0.095	3.43 ± 0.146	0.002	0.620	0.448
*Plasma lipid profile*							
Total cholesterol (mg/l)	863 ± 49.5	884 ± 77.3	892 ± 46.6	837 ± 103.5	0.903	0.818	0.607
HDL-cholesterol (mg/l)	408 ± 17.5	393 ± 25.7	366 ± 14.4	353 ± 41.0	0.139	0.606	0.971
LDL-cholesterol (mg/l)	83.1 ± 6.41	84.9 ± 12.70	78.0 ± 6.46	84.0 ± 9.45	0.745	0.672	0.820
VLDL-cholesterol (mg/l)	35.0 ± 4.59	35.2 ± 2.07	34.0 ± 2.42	36.8 ± 2.98	0.925	0.640	0.685
Triacylglycerols^†^ (mg/l)	175 ± 23.0	176 ± 10.3	170 ± 12.1	184 ± 14.9	0.925	0.640	0.685
Non esterified fatty acids (mM)	0.06 ± 0.02	0.06 ± 0.01	0.06 ± 0.02	0.03 ± 0.01	0.219	0.250	0.447
Total lipids (mg/l)	3401 ± 113.0	3444 ± 160.0	3454 ± 96.6	3358 ± 209.3	0.914	0.862	0.650
*Plasma hepatic markers*							
AST (U/l)	83.0 ± 3.60	99.7 ± 15.50	71.3 ± 3.24	67.2 ± 3.74	0.021	0.464	0.236
ALT (U/l)	30.4 ± 1.83	28.9 ± 3.02	26.5 ± 2.21	18.1 ± 1.69	0.003	0.036	0.136
ALP (U/l)	199 ± 37.2	173 ± 25.8	218 ± 25.1	200 ± 25.7	0.430	0.461	0.902

^†^Published in Costa *et al*. [12].

### Total lipids and fatty acid composition analysis

The detailed fatty acid composition of the subcutaneous and mesenteric adipose tissues, as well as that of *longissimus lumborum* muscle, was published in companion papers (Costa *et al*. [12,13], respectively). The liver total fatty acid content and composition is depicted in Table 2. Total fatty acids content was higher in the liver from Barrosã when compared to Alentejana bulls (*P* < 0.01). There were breed determined differences in 5 of the 31 identified fatty acids, but diet had the most important role over the individual fatty acid percentages. Alentejana bulls showed consistently lower percentages of 18:1 *t*11, *c*9, *t*11-CLA, 20:3n-9, 20:1*c*11 and TFA, when compared to the Barrosã bulls, but higher 20:4n-6 and 22:6n-3 percentages (*P* < 0.05). The high silage diet promoted the deposition of 14:1*c*9, 15:0, 18:3n-3, 20:5n-3, 22:5n-3, 22:6n-3 and 23:0 when compared to the low silage diet (*P* < 0.05). In contrast, the percentages of 18:1 *t*6-*t*8, 18:1 *t*9, 18:1 *t*10, 18.1 *t*12, 18:1*c*11, 18:1*c*13 and 22:4n-6 were higher in the low silage fed bulls, when compared to those fed the high silage diet (*P* < 0.05). Total n-3 PUFA and n-3 LC-PUFA were higher in the liver from the high silage fed bulls, when compared to those fed the low silage diet (*P* < 0.001). The Barrosã bulls fed the low silage had the lowest total PUFA and n-6 PUFA percentages (breed × diet, *P* < 0.05).

**Table 2:** **Total fatty acids and fatty acid composition of liver from Alentejana and Barrosã bulls fed high or low silage diets**^**1-3**^

	**Alentejana**				**Barrosã**				***P *** **value**		
	**HS**	**SE**	**LS**	**SE**	**HS**	**SE**	**LS**	**SE**	**Breed**	**Diet**	**Breed × Diet**
*Total fatty acids*	1.98	0.02	1.98	0.02	2.05	0.02	2.04	0.03	0.007	0.816	0.858
*Fatty acids*											
14:0	0.49	0.04	0.46	0.05	0.51	0.05	0.58	0.07	0.204	0.701	0.425
14:1*c*9	0.29	0.02	0.20	0.05	0.28	0.02	0.19	0.02	0.795	0.008	0.852
15:0	0.23	0.01	0.18	0.02	0.22	0.02	0.21	0.01	0.573	0.039	0.229
16:0	9.59	0.45	9.43	0.55	9.42	0.32	11.35	0.90	0.154	0.150	0.091
16:1*c*7	0.23	0.01	0.25	0.02	0.24	0.01	0.30	0.05	0.266	0.192	0.494
16:1*c*9	0.53	0.07	0.54	0.07	0.55	0.05	0.78	0.12	0.126	0.161	0.163
17:0	1.08	0.02	1.05	0.05	1.07	0.05	0.96	0.04	0.244	0.084	0.311
17:1*c*9	0.31	0.03	0.29	0.02	0.29	0.01	0.31	0.03	0.910	0.907	0.555
18:0	33.66	0.49	33.57	0.96	33.24	0.51	33.16	1.09	0.614	0.919	0.997
18:1 *t*6-*t*8	0.05	0.00	0.07	0.01	0.05	0.00	0.06	0.00	0.647	0.010	0.922
18:1 *t*9	0.06	0.00	0.08	0.00	0.06	0.00	0.09	0.01	0.333	<0.001	0.382
18:1 *t*10	0.07	0.00	0.16	0.02	0.07	0.00	0.11	0.01	0.068	<0.001	0.060
18:1 *t*11	0.86	0.07	0.97	0.09	1.09	0.11	1.11	0.07	0.049	0.484	0.644
18:1 *t*12	0.37	0.02	0.43	0.03	0.37	0.01	0.44	0.03	0.794	0.019	0.792
18:1*c*9	11.42	0.69	10.86	0.52	11.11	0.30	12.91	0.89	0.186	0.345	0.078
18:1*c*11	1.58	0.06	1.78	0.13	1.55	0.05	1.99	0.20	0.516	0.022	0.370
18:1*c*12	0.28	0.02	0.27	0.02	0.25	0.02	0.31	0.03	0.775	0.224	0.106
18:1*c*13	0.10	0.01	0.13	0.01	0.10	0.01	0.13	0.01	0.964	0.010	0.792
18:1 *t*16 + *c*14	0.10	0.01	0.10	0.01	0.09	0.00	0.11	0.01	0.873	0.567	0.197
18:2n-6	16.16	0.87	17.08	1.17	16.32	0.51	14.67	1.02	0.235	0.694	0.174
18:3n-3	1.24	0.08	0.80	0.06	1.21	0.05	0.73	0.06	0.465	<0.001	0.796
CLA (*c*9*t*11)	0.37	0.02	0.28	0.03	0.48	0.04	0.44	0.06	0.001	0.098	0.453
20:0	0.12	0.01	0.13	0.02	0.13	0.01	0.14	0.01	0.389	0.363	0.859
20:1*c*11	0.09	0.01	0.11	0.01	0.13	0.01	0.12	0.01	0.010	0.466	0.273
20:2n-6	0.30	0.03	0.30	0.04	0.30	0.02	0.25	0.03	0.362	0.446	0.312
20:3n-9	0.39	0.03	0.33	0.03	0.77	0.04	0.78	0.04	<0.001	0.462	0.336
20:3n-6	2.43	0.21	2.76	0.29	2.54	0.25	2.64	0.25	0.982	0.383	0.636
20:4n-6	9.24	0.18	9.39	0.23	9.00	0.19	7.96	0.56	0.022	0.196	0.088
20:5n-3	0.42	0.02	0.25	0.02	0.37	0.02	0.26	0.02	0.412	<0.001	0.279
22:4n-6	1.64	0.10	2.26	0.21	1.86	0.12	1.97	0.22	0.839	0.042	0.145
22:5n-3	2.93	0.14	2.60	0.14	3.03	0.07	2.11	0.21	0.204	<0.001	0.056
22:6n-3	1.28	0.10	0.79	0.06	1.06	0.05	0.70	0.08	0.044	<0.001	0.393
23:0	0.21	0.04	0.13	0.02	0.22	0.02	0.16	0.02	0.494	0.009	0.753
Other^4^	1.90	0.06	1.99	0.10	2.01	0.08	2.01	0.10	0.435	0.604	0.606
*Partial sums*											
SFA	45.38	0.24	44.95	0.63	44.82	0.25	46.55	0.96	0.393	0.292	0.087
MUFA	14.83	0.83	14.43	0.75	14.50	0.38	17.03	1.28	0.205	0.231	0.106
TFA	1.89	0.11	2.07	0.15	2.21	0.16	2.36	0.12	0.033	0.233	0.896
PUFA	36.01^b^	0.83	36.56^b^	0.85	36.46^b^	0.37	32.05^a^	1.55	0.055	0.066	0.021
n-3 PUFA	5.87	0.25	4.43	0.20	5.67	0.12	3.79	0.31	0.082	<0.001	0.349
n-6 PUFA	29.75^ab^	0.77	31.80^b^	0.82	30.02^ab^	0.31	27.48^a^	1.30	0.030	0.778	0.015
n-3 LCPUFA	4.63	0.24	3.64	0.22	4.46	0.12	3.06	0.28	0.107	<0.001	0.368
n-6 LCPUFA	13.59	0.30	14.72	0.57	13.70	0.42	12.81	0.83	0.124	0.835	0.087

SFA = sum of 14:0, 15:0, 16:0, 17:0, 18:0, 20:0 and 23:0; MUFA = sum of 14:1c9, 16:1c7, 16:1c9, 17:1c9, 18:1c9, 18:1c11, 18:1c12, 18:1c13, and 20:1c11; TFA = sum of 18:1 t6-t8, 18:1 t9, 18:1 t10, 18:1 t11, 18:1 t12, 18:1 t16 + c14 and 18:2c9t11; PUFA = sum of 18:2n-6, 18:3n-3, 20:2n-6, 20:3n-9, 20:3n-6, 20:4n-6, 20:5n-3, 22:4n-6, 22:5n-3 and 22:6n-3.

^1^Means in the same row with different superscripts are significantly different (P < 0.05).

^2^Total fatty acids are expressed as g/100 g liver; fatty acid composition is expressed as mol% fatty acids.

^3^HS: high silage; LS: low silage.

^4^Other = sum of i-15:0, a-15:0, i-16:0, i-17:0, a-17:0 and dimethylacetals (16:0 DMA and 18:0 DMA).

### Gene expression analysis

**Figure 1:**
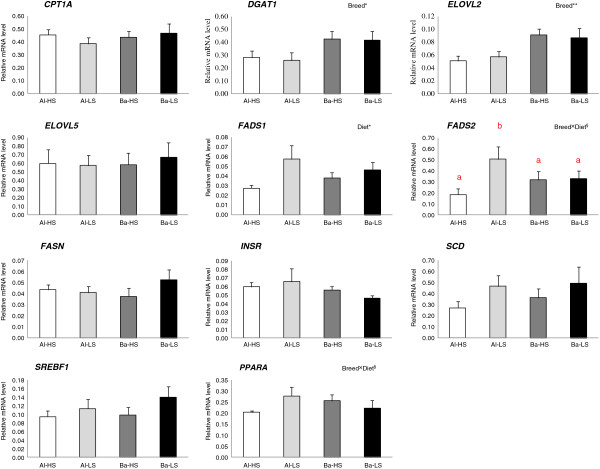
**Relative expression levels of eleven selected genes in the liver from Alentejana and Barrosã bulls fed high or low silage diets.** Each value was normalized to *RPS9* and *SDHA* expression. Al-HS: Alentejana bulls fed the high silage diet; Al-LS: Alentejana bulls fed the low silage diet; Ba-HS: Barrosã bulls fed the high silage diet; Ba-LS: Barrosã bulls fed the low silage diet. Error bars indicate standard error. ^†^Tendencies were considered for 0.05 < *P* < 0.10. ^a,b,c^Least square means with different superscripts differ at least *P* < 0.05.

Results from the gene expression analysis are shown in Figure 1. Breed influenced the mRNA levels of both *DGAT1* and *ELOVL2*, with higher values for the Barrosã when compared to Alentejana bulls (*P* < 0.05 and *P* < 0.01, respectively). In turn, the expression levels of *FADS1* were higher in low silage in comparison to high silage fed bulls (*P* < 0.05). Similarly, the low silage diet tended to promote higher *FADS2* mRNA expression levels than the high silage diet, but only in the Alentejana bulls (breed × diet *P* = 0.072). In addition, Alentejana bulls tended to have the highest *PPARA* gene expression levels when fed the low silage diet, whereas the inverse trend was found for Barrosã bulls (breed × diet, *P* = 0.086). Neither breed nor diet influenced the expression of *CPT1A*, *ELOVL5*, *FASN*, *INSR*, *SCD* and *SREBF1* genes (*P* > 0.05).

### Correlation analysis

The correlation analysis between genes and fatty acid percentages is depicted in Table 3. The *FADS1* and *FADS2* genes were shown as the most associated with fatty acid composition, along with *CPT1A* and *ELOVL5*. Positive correlations were found for the *CPT1A* expression level and the 14:0 (*r* = 0.56), and 16:0 (*r* = 0.60) percentages. The *CPT1A* gene was also positively correlated with the percentages of 16:1*c*9 (*r* = 0.47), 17:1*c*9 (*r* = 0.31), and 18:1*c*9 (*r* = 0.42). Negative correlations were observed between the *CPT1A* gene and 20:2n-6 (*r* = -0.59) and 22:4n-6 (*r* = -0.32) fatty acids.

**Table 3:** **Pearson correlation coefficients between the fatty acid composition and the relative gene expression levels in the liver from Alentejana and Barrosã bulls fed high or low silage diets**^**1−4**^

	***CPT1A***	***DGAT1***	***ELOVL2***	***ELOVL5***	***FADS1***	***FADS2***	***FASN***	***INSR***	***PPARA***
14:0	0.56***			0.40*	0.39*	0.34*		0.32*	
14:1*c*9							0.42**		
16:0	0.60***		0.44**	0.60***	0.42**				
16:1*c*7					0.48**	0.44**			
16:1*c*9	0.47**		0.37*	0.51***	0.38*				
17:0			−0.37*						
17:1*c*9	0.31*			0.39*	0.33*				
18:0				−0.40**	−0.41**				
18:1*t*6*t*8						0.32*			
18:1 *t*9					0.41*	0.40*			
18:1 *t*10		−0.34*							
18:1 *t*11									
18:1 *t*12									
18:1*c*9	0.42**		0.33*	0.50**	0.38*				
18:1*c*11					0.51**	0.40*			
18:1*c*12									
18:1*c*13					0.48**	0.39*			
18:1*t*16*c*14									
18:2n-6				−0.32*	−0.53***	−0.52***		−0.33*	
20:0					0.48**	0.68***			
18:3n-3					−0.46**	−0.50**			
20:1*c*11						0.38*		0.32*	0.38*
20:2n-6	−0.59***			−0.46**	−0.34*			−0.38*	
20:3n-9		0.44**	0.47**						
20:3n-6					0.50**	0.67***		0.35*	
20:4n-6									0.34*
23:0									
20:5n-3									
22:4n-6	−0.32*				0.72***	0.70***			
22:5n-3								0.32*	

^1^Statistical significance of Pearson correlation coefficients: *, P < 0.05; **, P < 0.01; ***, P < 0.001.

^2^Fatty acid contents expressed as mol/g liver.

^3^There were no significant correlations between *SCD* or *SREBF1* and individual fatty acids.

^4^There were no significant correlations between 15:0, CLA (c9t11), 22:6n-3 and the mRNA levels of the genes under analysis.

The expression levels of *DGAT1* showed a moderate positive correlation with the 20:3n-9 percentage (*r* = 0.44). The percentages of 16:0 (*r* = 0.44), 16:1*c*9 (*r* = 0.37) and 20:3n-9 (*r* = 0.40) showed positive correlations with the *ELOVL2* gene expression levels. A negative correlation was observed between *ELOVL2* mRNA level and the 17:0 percentage (*r* = -0.37). Expression of the *ELOVL5* gene was positively correlated with the percentages of 14:0 (*r* = 0.40), 16:0 (*r* = 0.60), 16:1*c*9 (*r* = 0.51), 17:1*c*9 (*r* = 0.39) and 18:1*c*9 (*r* = 0.50). In addition, there were *ELOVL5* mRNA levels were negatively correlated with 18:0 (*r* = -0.40), 18:1*c*9 (*r* = 0.33), 18:2n-6 (*r* = -0.32) and 20:2n-6 (*r* = -0.46).

The *FADS1* mRNA levels were positively associated with 14:0 (*r* = 0.39), 16:0 (*r* = 0.42), 16:1*c*7 (*r* = 0.48), 16:1*c*9 (*r* = 0.38), 17:1*c*9 (*r* = 0.33), 18:1*c*9 (*r* = 0.38), 18:1*c*11 (*r* = 0.51), 18:1*c*13 (*r* = 0.48), 20:0 (*r* = 0.48), 20:3n-6 (*r* = 0.50) and 22:4n-6 (*r* = 0.72) fatty acids. In addition, *FADS1* showed negative correlations with 18:0 (*r* = -0.41), 18:2n-6 (*r* = -0.53), 18:3n-3 (*r* = -0.46) and 20:2n-6 (*r* = -0.34). The relative expression of *FADS2* was positively correlated with the percentages of 14:0 (*r* = 0.34), 16:1*c*7 (*r* = 0.44), 18:1 *t*9 (*r* = 0.40), 18:1*c*11 (*r* = 0.40), 18:1*c*13 (*r* = 0.39), 20:0 (*r* = 0.68), 20:1*c*11 (*r* = 0.38), 20:3n-6 (*r* = 0.67) and 22:4n*-*6 (*r* = 0.70). Negative correlations were found between the *FADS2* and 18:n-6 (*r* = -0.52) and 18:3n-3 (*r* = -0.50) fatty acids percentages. The 14:1*c*9 fatty acid was positively correlated with the *FASN* gene expression levels (*r* = 0.42).

The *INSR* relative mRNA levels were positively correlated with the 14:0 (*r* = 0.32), 20:1*c*11 (*r* = 0.32), 20:3n-6 (*r* = 0.35), 22:5n-3 (*r* = 0.32) percentages, but negatively associated with the 18:2n-6 (*r* = -0.33) and 20:2n-6 percentages (*r* = -0.38). The PPARA mRNA levels showed positive correlations with 20:1c11 (*r* = 0.38) and 20:4n-6 (*r* = 0.34) fatty acids percentages.

### Principal components analysis

**Figure 2:**
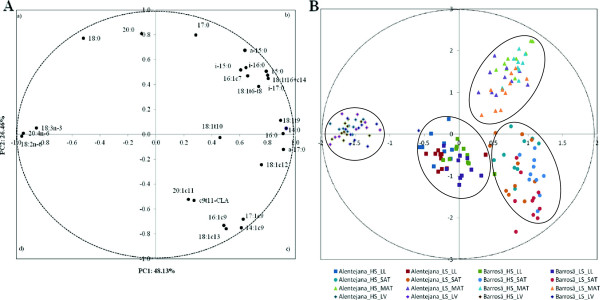
**Loading plot of the first and second principal components of the pooled data (A) and component’s score vectors (B) for *longissimus lumborum *muscle, subcutaneous adipose tissue, mesenteric adipose tissue and liver from Alentejana and Barrosã bulls fed high or low silage diets.** LL: *longissimus lumborum* muscle; SAT: subcutaneous adipose tissue; MAT: mesenteric adipose tissue; LV: liver; Alentejana -HS: Alentejana bulls fed the high silage diet; Alentejana-LS: Alentejana bulls fed the low silage diet; Barrosã -HS: Barrosã bulls fed the high silage diet; Barrosã -LS: Barrosã bulls fed the low silage diet.

A PCA of the fatty acid composition of muscle, subcutaneous adipose tissue, mesenteric adipose tissue and liver was performed in order to assess the distinctive metabolic features of each tissue and to describe the variability of the pooled data into two dimensions (Figure 2A). The score plot of the first two components explains 74.59% of the total variability, with 48.13% for PC1 and 26.46% for PC2 (Table 4). The score plot from PCA showed the fatty acids associated into four main clusters. In quadrant *b)*, a cluster is formed by BCFA (*a*-15:0, *i*-15:0, *i*-16:0, *i*-17:0) and some 18:1 isomers (18:1-*t*16 and 18:1-*c*14 co-eluted, and 18:1-*t*6 to *t*8). A second cluster, with a significant contribution to PC1, was formed by 14:0, 16:0, *a*-17:0, 18:1-*t*9 and 18:1-*t*12. In quadrant *d)*, a cluster was formed by *c*9*t*11-CLA and some MUFA (14:1*c*9, 16:1*c*9, 17:1*c*9, 18:1*c*11 and 20:1*c*11). Finally, 18:2n-6, 18:3n-3 and 20:4n-6 formed a cluster in quadrant *c)*, with little or no contribution to PC2 but with significant impact on PC1.

**Table 4:** **Loadings for the first three principal components**^**1**^

**Variable**	**PC1**	**PC2**	**PC3**
14:0	0.935	0.049	−0.082
*i*-15:0	0.659	0.470	0.447
*a*-15:0	0.639	0.675	0.182
14:1c9	0.488	−0.731	0.190
15:0	0.791	0.507	0.034
*i*-16:0	0.645	0.537	0.391
16:0	0.913	0.006	−0.178
*i*-17:0	0.806	0.449	0.160
16:1*c*7	0.608	0.520	0.161
16:1*c*9	0.612	−0.748	0.108
*a*-17:0	0.915	−0.121	0.217
17:0	0.287	0.800	−0.201
17:1*c*9	0.626	−0.680	−0.088
18:0	−0.520	0.772	0.071
18:1 *t*6-*t*8	0.738	0.386	−0.366
18:1 *t*9	0.895	0.112	−0.198
18:1 *t*10	0.460	−0.026	−0.718
18:1 *t*11	0.612	0.531	0.108
18:1*c*9	0.763	−0.560	−0.063
18:1*c*11	0.684	−0.581	−0.020
18:1*c*12	0.757	−0.243	−0.096
18:1*c*13	0.503	−0.757	0.032
18:1 *t*16 + *c*14	0.801	0.476	0.124
18:2n-6	−0.961	−0.015	0.040
20:0	−0.101	0.809	−0.063
18:3n-3	−0.858	0.051	0.225
20:1c11	0.233	−0.521	0.185
*c*9*t*11-CLA	0.272	−0.530	0.566
20:4n-6	−0.951	0.009	0.134
Proportion of variance (%)	48.13	26.46	0.06
Cumulative variance (%)	48.13	74.59	80.73

^1^PC: principal component.

The score plot depicted in Figure 2B showed the location of the four tissues in the multivariate space of the first two PCs. These scores were notably arranged in four clusters, corresponding to the four tissues analysed. The most notable result from this statistical approach is the higher distance between the cluster formed by the liver and the remaining three clusters.

## Discussion

The present study was based on an experiment with two genetically different bovine breeds with distinct maturity rates, Alentejana and Barrosã, fed diets with different silage to concentrate ratio (30/70% *vs.* 70/30%). Alentejana and Barrosã breeds, despite being phylogenetically distant [14] share more genetic similarities than the breeds used in previous studies addressing the differences between breeds in fatty acid metabolism, mainly based on the Japanese Black and Holstein breeds [15-17]. We observed in these animals that different fat depots, subcutaneous and mesenteric adipose tissues, had distinct features regarding cellularity and fatty acid composition [12]. Results indicated that genetic background and, to a lesser extent diet composition, determine fat content and composition, pointing out to a differential fat partitioning between subcutaneous and intramuscular fat in Alentejana and Barrosã breeds. In addition, the comparison of fatty acid composition and gene expression levels between the muscle and subcutaneous adipose tissue indicated that these tissues play distinct roles in lipid metabolism, reinforcing the prevailing role of the subcutaneous adipose tissue over intramuscular fat in the *de novo* fatty acid synthesis (data not shown). In fact, the PCA showed a clear separation of the liver from the remaining tissues (muscle, and subcutaneous and mesenteric adipose tissues), to which contributed the fact that this tissue is particularly rich in α-linolenic acid and the products of its desaturation and elongation, the n-3 LC-PUFA. This result suggests that the liver may have an important role in the n-3 LC-PUFA biosynthesis.

The major metabolic fates of long chain fatty acid CoA in the liver are: i) esterification into triacylglycerols and, to a lesser extent, into phospholipids and cholesterol esters, ii) complete oxidation to CO_2_ or incomplete oxidation which generates acetate and ketone bodies [1]. De La Torre *et al*. [18] reported that, once incorporated *in vitro* into bovine hepatic cells, rumenic and oleic acids are highly catabolised through the β-oxidation pathway. One possible explanation might be the low efficiency of bovines in secreting fatty acids from the liver [19], thus directing fatty acids preferentially towards the oxidative pathway.

The liver is responsible for the uptake of NEFA and subsequent storage as triacylglycerols or release as VLDL. Hepatic triacylglycerols synthesis is the result of various pathways of lipid metabolism, including fatty acid uptake from plasma, oxidation of fatty acids, *de novo* synthesis of fatty acids and secretion of triacylglycerols via VLDL [20,21]. Apart from hormones, the metabolic regulatory events in the liver are directed by metabolites levels, either in excess (*e.g.*, NEFA) or in shortage (*e.g.*, glucose) [22]. Once in the hepatocytes, catabolism of fatty acids is mostly directed towards the synthesis of ketone bodies for energy utilisation by tissues [4,23]. In spite of the higher insulin levels observed in the bulls fed the low silage diet (data not shown), when compared to those fed the high silage diet, it had no impact on *INSR* mRNA levels in the liver. Zhang *et al*. [24] reported low *INSR* gene expression levels in calf cultured hepatocytes in response to high insulin concentrations. However, it should be noted that in the present study even the highest insulin levels were within physiological levels.

Due to gluconeogenesis in liver, both propionate and lactate can indirectly modulate adipose tissue lipogenesis through increased glucose availability. The glucogenic effect of high-starch diets is often accompanied by an enhanced insulin response [25]. The diacylglycerol acyltransferase (DGAT) catalyses the final step in triacylglycerol biosynthesis by converting diacylgycerols and fatty acyl-coenzyme A (CoA) into triacylglycero1s [26]. The *DGAT1* gene has also been related to increased hepatic triacylglycerol synthesis in dairy cows [27]. Moreover, mRNA levels of this gene appeared to be susceptible to diet-induced hyperinsulinaemia pre-partum [27]. In the present study, the relative expression levels of *DGAT1* was higher in the liver from Barrosã bulls, when compared to Alentejana bulls, although plasma insulin was affected by diet and not by breed as reported in a companion paper [12].

It was suggested that bovine liver converts more efficiently linoleic acid into arachidonic acid than α-linolenic acid into n-3 LC-PUFA [22]. In line with this, in the present study the ARA acid percentages were the highest amongst LC-PUFA. The PCA from the fatty acid pooled data showed a clear separation of the tissues analysed, with particular emphasis on the liver. The LA and α-linolenic acid, along with ARA acid, were identified as the most contributing for the distancing of the liver cluster from those of the remaining tissues. This observation could be explained by the higher phospholipid/triacylglycerol ratio in the liver when compared to the other tissues. Given that LA and α-linolenic acid originate from diet, this suggests that the liver plays a role in their metabolism, possibly through their desaturation and elongation.

Attending to the putative role of liver in fatty acid elongation and desaturation, as described by others [4,11] and as suggested by the results herein presented for hepatic fatty acid composition, this work was focused on the analysis of gene expression levels of enzymes and transcription factors associated with the n-3 and n-6 LC-PUFA pathway synthesis. The PPARA, a transcription factor that acts as an important regulator of lipid metabolism and energy homeostasis, plays a key role in the control of the pathways involved in fatty acid uptake, fatty acid binding, fatty acid oxidation, ketogenesis, as well as carnitine synthesis [28]. The fact that the mRNA level of *CPT1A*, which is controlled by *PPARA*, was unchanged across experimental groups could indicate that there was no activation of *PPARA* in the liver. Moreover, activation of *PPARA* is known to be caused by increased plasma concentrations of NEFA. However, in the present study, there were no significant changes in NEFA plasma levels, which is consistent with the similar *PPARA* levels across experimental groups.

Polyunsaturated fatty acids that escape β-oxidation could be converted into longer and/or more unsaturated fatty acids by the elongation-desaturation pathway, LA and α-linolenic acid being metabolised, theoretically, into 20:4n-6 and 22:6n-3, respectively [29,30]. In a recent study by Gruffat *et al*. [4], the conversion of α-linolenic acid into longer and/or more unsaturated fatty acids was not detected, yet 13.5% of LA was converted into ARA. It is widely accepted that members of the n-6 and n-3 families compete for the elongation-desaturation pathway [31]. Furthermore, the conversion of α-linolenic acid and LA into their longer chain homologues is greatly determined by the composition of dietary fats [32]. Harnack *et al*. [33] suggested that a ratio of 1/1 would lead to the highest formation of n-3 LC-PUFA, given that the conversion of n-3 fatty acids into higher homologues may depend on the ratio of ingested n-6/n-3 fatty acids.

The carnitine palmitoyltransferase (CPT) system is an essential step in the β-oxidation of fatty acids, including LC-PUFA. Gruffat *et al*. [4] showed that the rate of α-linolenic acid oxidation was higher than that of LA, possibly due to higher mitochondrial activity of CPT1A, as suggested by Ide *et al*. [34]. The positive correlations between *CPT1A* mRNA levels and the main SFA and MUFA, as well as the negative relationship with some PUFA (20:2n-6 and 22:4n-6) may be attributed to the role of the enzyme in fatty acid β-oxidation and ketogenesis. The higher SFA and MUFA percentages are a consequence of increased triacylglycerol and NEFA in the hepatic tissue, which would in turn promote fatty acid β-oxidation in order to prevent their excessive accumulation.

*SREBF1* is a fundamental regulator of fatty acid biosynthesis, suggesting that it could be a key point of control of membrane lipid homeostasis capable of strongly influencing the lipid composition of membranes [35]. The *SREBF1* gene regulates a wide array of genes involved in lipid biosynthesis. In the present work, the *SREBF1* expression was shown to be correlated with the mRNA levels of *FASN*, *SCD*, *PPARA* and *INSR* (data not shown). The fatty acid synthase, encoded by *FASN*, plays a central role in *de novo* lipogenesis in mammals. However, the *FASN* mRNA levels were similar across experimental groups and showed no correlation with the main liver fatty acids, thus suggesting no modulation by diet or silage level under these experimental conditions.

The *SCD* gene encodes for an important enzyme in unsaturated fatty acid synthesis [36]. SCD activity in bovine liver and duodenal mucosal cells has been reported [37-39]. Furthermore, Chung *et al*. [39] suggested that some portion of the MUFA in adipose tissues may arise from hepatic and mucosal desaturation of dietary SFA. However, in the present work, there was no apparent association between *SCD* gene expression levels and the desaturation indices. This finding reinforces the concept of a low SCD activity in the liver, which is not to say that there is no desaturation at all. The matter of fact is that hepatic desaturation activity seems to be carried out mainly by the enzymes encoded by *FADS1* and *FADS2*.

The higher expression levels of *FADS1* and *FADS2* in the low silage than in the high silage fed bulls, particularly Alentejana bulls, is concomitant with the diet effect observed in the percentages of most LC-PUFA and, therefore, with the correlations found. That is, feeding the low silage diet, poorer in 18:2n-6 and α-linolenic acid than the high silage diet, promoted the expression of the genes encoding Δ5 and Δ6 desaturases in order to incorporate an adequate level of LC-PUFA in membrane phospholipids. The increase in the *FADS1* gene expression could have contributed to promote lipid biosynthesis and fatty acid deposition. The *FADS1* gene has been considered as one of the rate-limiting enzymes to the endogenous formation of LC-PUFA in humans [40]. In mammals, FADS1 converts dihomo-γ-linolenic acid (20:3n-6) to ARA and eicosatetraenoic acid (20:4n-3) to EPA with, respectively, LA and α-linolenic acid as the initial substrates [40,41].

Liver seems to be highly active in α-linolenic acid catabolism [18], thus limiting its subsequent availability for deposition in muscles. There are two basic metabolic fates for α-linolenic acid. First, it is subjected to β-oxidation and extensive carbon recycling. Second, it is converted into longer fatty acids via the elongation and desaturation pathway. Elongation of C18 in the LC-PUFA pathway occurs through elongase enzymes, which have been suggested to have a regulatory role on LC-PUFA synthesis and may be transcribed from one or more genes (*ELOVL2* and *ELOVL5*) [42]. Hepatic LC-PUFA metabolism has been linked to energy balance and physiological state in dairy cattle. Long-chain fatty acyl elongases (ELOVL) are endoplasmatic reticulum membrane-bound proteins responsible for the first regulatory step in the fatty acid elongation pathway (condensation of activated fatty acids with malonyl-CoA), elongating fatty acid that are biosynthesized *de novo* or supplied by the diet [43]. In mammals, ELOVL2 has greatest activity in the elongation of C20 and C22 but low or, in the case of humans, no activity towards C18 PUFA [44]. In contrast, mammalian ELOVL5 is very active towards C18 PUFA but does not appear to have the capacity to elongate beyond C22 [44,45]. Cherfaoui *et al*. [11] proposed that the limiting step for elongation of α-linolenic acid in the muscle tissues is the absence of ELOVL5 protein. Moreover, these authors also suggested that the low levels of DHA in the muscle tissues could be a consequence of its preferential peroxidation or of its preferential uptake by other tissues.

Hepatic *ELOVL2* and *ELOVL5* are both regulated by *SREBP* transcription factors in mouse [46]. Nonetheless, in the present study there seemed to be no direct correlation between *SREBF1* mRNA levels and the expression of *ELOVL2* and *ELOVL5* genes, in spite of *SREBF1* being correlated with total n-6 PUFA. The higher *ELOVL2* gene expression, and thus the higher fatty acid elongase with high activity in the final steps of LC-PUFA biosynthesis, in the Barrosã than in the Alentejana bulls could provide a route to promote EPA and DHA deposition. However, LC-PUFA contents in the liver from the present study were influenced, not by breed, but by diet.

## Conclusion

In summary, the present study suggests that liver has, among the bovine lipogenic tissues, a specific role in lipid metabolism. In addition, the results indicate a breed modulation of hepatic desaturation/elongation of fatty acids, possibly through the differential expression of genes encoding for enzymes involved in the desaturation and elongation pathway. In some cases, the response to varying silage levels was modulated by the genetic background (*FADS2* and *PPARA*), whereas in others (*DGAT1* and *ELOVL2*) there was a clear breed effect regardless of diet composition. The small differences observed in gene expression levels could have additive effects, which may explain the differences found in the hepatic fatty acid profile, particularly in the LC-PUFA. In summary, the results herein discussed are in line with the previous reports from this experiment, thus stressing the importance of devising custom-made feeding strategies which take into account the genetic background.

## Methods

### Animals and experimental diets

This trial was conducted at the facilities of Unidade de Produção Animal, L-INIA, INIAV (Fonte Boa, Vale de Santarém, Portugal), from January to November 2009. A Animals were handled in accordance with local and national guidelines covering animal experiments, reviewed by the Ethics Commission of CIISA/FMV and approved by the Animal Care Committee of the National Veterinary Authority (Direccão-Geral de Veterinária), following the appropriate European Union guidelines (Directive 86/609/EEC).

Forty young bulls from Alentejana (*n* = 20) and Barrosã (*n* = 20), were assigned to high or low forage based diets (four experimental groups of 10 animals each). One Alentejana bull from the high silage fed group was later removed from the trial due to a limp. Diets were composed of 30/70% (low silage) and 70/30% (high silage) of maize silage and concentrate, respectively. The detailed proximate and fatty acid composition of the experimental diets has been published in a previous paper [47]. Briefly, crude fat and starch were higher in the low silage (31.7 and 376 g/kg DM, respectively) than in the high silage diet (28.7 and 285 g/kg DM, respectively). Conversely, the high silage diet had higher crude fibre and NDF contents (198 and 403 g/kg DM, respectively) in comparison to the low silage diet (150 and 321 g/kg DM, respectively). The low silage diet had lower palmitic (20.2 *vs*. 24.1%) and stearic acid (5.1 *vs*. 9.4%) percentages than the high silage diet, while the latter showed higher proportions of 20:0 (6.5 *vs*. 3.7%), 18:2n-6 (43.9 *vs*. 40.9%) and 18:3n-3 (9.2 *vs*. 6.0%). Animals were housed in eight adjacent pens, two pens per breed and diet. The initial age was 331 ± 32 days for Alentejana bulls and 267 ± 10 days for Barrosã bulls. All animals were slaughtered at 18 months old, which is a common commercial slaughter age for bulls in Portugal. Slaughters were performed at the INIAV experimental abattoir by exsanguination, after stunning with a cartridge-fired captive bolt stunner.

### Blood sampling

One week prior to slaughter, blood samples were collected from the tail vein and centrifuged (3000 rpm for 15 minutes at room temperature) to harvest heparinized plasma. Total cholesterol, HDL-cholesterol, LDL-cholesterol, triacylglycerols, aspartate aminotransferase (AST), alanine aminotransferase (ALT) and alkaline phosphatase (ALP) were analysed using diagnostic test kits (Roche Diagnostics, Mannheim, Germany), in a Modular Hitachi Analytical System (Roche Diagnostics). VLDL-cholesterol and total lipids were calculated as described by Friedewald *et al*. [48] and Covaci *et al*. [49] formulas, respectively.

Plasma non-esterified fatty acids (NEFA) were quantified using the Free Fatty Acid Quantification Kit (Biovision Inc, Mountain View, CA, USA).

### Sample collection

Immediately after slaughter, liver samples for gene expression analysis were collected, rinsed with sterile RNAse-free water solution, cut into small pieces (thickness of ~0.3 cm), stabilised in RNA Later solution (Qiagen, Hilden, Germany) and subsequently stored at –80°C. A second sample (approximately 50 g) was vacuum-packed and stored at –20°C, until lipid extraction and determination of fatty acid composition.

### Total lipid content and fatty acid composition

Liver samples were lyophilised (-60°C and 2.0 hPa), maintained exsiccated at room temperature and analysed within two weeks. Total lipids were extracted by the method of Folch *et al.*[50], using dichloromethane and methanol (2:1 v/v) instead of chloroform and methanol (2:1 v/v), as modified by Carlson [51].

Fatty acids were then converted to methyl esters as described by Raes *et al*. [52], using sodium methoxide in anhydrous methanol (0.5 mol/L) for 30 min, followed by hydrochloric acid in methanol (1:1 v/v) for 10 min at 50°C. Fatty acid methyl esters (FAME) were extracted twice with 3 mL of *n*-hexane and pooled extracts were evaporated at 35°C, under a stream of nitrogen, until a final volume of 2 mL. The resulting FAME were then analysed by GC using a fused-silica capillary column (CP-Sil 88; 100 m × 0.25 mm i.d., 0.20 mm film thickness; Chrompack, Varian Inc., Walnut Creek, CA, USA), equipped with a flame ionisation detector, as described by Bessa *et al.*[9]*.* The quantification of FAME used nonadecanoic acid (19:0) as the internal standard, added to lipids prior to saponification and methylation. Fatty acid composition was calculated assuming a direct relationship between peak area and fatty acid methyl ester weight.

### Total RNA isolation

Frozen tissue samples were homogenized with an Ultra-Turrax® homogenizer (IKA-Labortechnik, Staufen, Germany). Total RNA was extracted from liver samples using Trizol reagent (Invitrogen, Carlsbad, CA, USA) and purified with the RNeasy Mini Kit (Qiagen Inc), according to the manufacturer’s protocol. To exclude possible amplification of contaminating genomic DNA, an additional step of DNase digestion was performed with the RNase-free DNase Set (Qiagen Inc.), incubating samples with DNase for 15 min at room temperature. Total RNA extracts were immediately analysed for quantity (OD260nm) and purity (OD260nm/OD280nm) (NanoDrop ND-2000c, Peqlab GmbH, Erlangen, Germany). RNA aliquots were stored at –80°C and until further analysis.

### Synthesis of complementary DNA

Single-stranded cDNA was synthesised using the High Capacity cDNA Reverse Transcription Kit (Applied Biosystems, Foster City, CA, USA) following the manufacturer’s protocol. Each 20 μl RT reaction contained 1200 ng of RNA template, 50 nM random RT Primer, 1 × RT buffer, 0.25 mM of each dNTPs, 3.33 U/μl multiscribe reverse transcriptase and 0.25 U/μL RNase inhibitor, at temperatures of 25°C for 10 min, 37°C for 120 min, and 85°C for 5 min cDNA aliquots were stored at –20°C. Total liver RNA was reverse transcribed using a High Capacity cDNA Reverse Transcription Kit (Applied BioSystems) according to manufacturer’s instructions.

### Primer design and housekeeping gene stability evaluation

Forward and reverse primers were optimally designed to cover exon–exon junctions to account for alternative splicing when possible (Table 5).

**Table 5:** **Specifications of oligonucleotides used for RT-qPCR**^**1-3**^

**Gene symbol**	**Full gene name**	**Acc. Number**^**1**^	**Primer pairs (5′-3′)**	**Amplicon length**
CPT1A	carnitine palmitoyltransferase 1A	XM_002699420.2	F: TTCTTCTGGGGTCTACGATTCC	119
			R: GATGTGCTTGCTGTCCCTCAG	
DGAT1	diacylglycerol O-acyltransferase 1	NM_174693.2	F: TTGGCAGGTAAGGCGGC	99
			R: GGGGGCGAAGAGGAAGTAGT	
ELOVL2	fatty acid elongase 2	NM_001083517.1	F: GTCTTCTTACATGATGACGCTGGT	72
			R: ATTGGCTTTTTCCGGTATGTCTGA	
ELOVL5	fatty acid elongase 5	NM_001046597.1	F: CCCTCTCGGTTGGTTGTATTTC	127
			R: GTGGTCCTTTTGGTGCTCTCTC	
FADS1	fatty acid desaturase 1	XM_002699285.2	F: GTGGGTGGACTTGGCCTG	103
			R: TGGGGCTTGTCTTCATGGTC	
FADS2	fatty acid desaturase 2	NM_001083444.1	F: CGGCAAGAAGAAGCTGAAATACCTG	92
			R: CTGCTCATCCCTTTGTATTTCCA	
FASN	fatty acid synthase	NM_001012669.1	F: ATGGCGTTCCACTCCTACTTCA	137
			R: CTCTCCTGCCACTGGGTCTC	
INSR	insulin receptor	XM_002688832.2	F:ACGCTGGTGGTGATGGAGTT	133
			R: TCTCTGCCGCCATCTGAATC	
PPARA	peroxisome proliferator-activated receptor alpha	NM_001034036.1	F: CCAACAACAACCCGCCTTT	125
			R: CGTCTTCTCGGCCATACACA	
SCD	stearoyl-CoA desaturase 9	NM_173959.4	F: CCATCAACCCCCGAGAGAAT	76
			R: AAGGTGTGGTGGTAGTTGTGGAA	
SREBF1c	sterol regulatory element binding transcription factor 1	NM_001113302.1	F: ATCTCTTGGAGCGAGCACTGA	115
			R: AGGTACCCCAGGGCATCTG	
RPS9^3^	ribosomal protein S9	NM_001101152.1	F: GAAGGTAATGCCCTGTTGCG	141
			R: CAGGCCCAGCTTGAAGACC	
SDHA^3^	succinate dehydrogenase complex subunit A	NM_174178.2	F: TGCAGGAAGGCTGTGAGAAGAT	100
			R: GTCTCCACCAGGTCAGTGTTCC	

^1^Entrez Gene, National Center for Biotechnology Information (NCBI).

^2^F: forward primer and R: reverse primer.

^3^Housekeeping gene.

Primer 3 (http://primer3.ut.ee/) and Primer Express software v3.1 (Applied Biosystems, Foster City, CA, USA) were used to design primers for candidate housekeeping (HKG) and target genes, amplicon length was fixed to 70–150 bp. When possible, primer sets were designed to fall across exon–exon junctions. Sequences, amplicon length and reference sequences are summarised in Table 5. Primers were aligned against publicly available databases using BLASTN suite at the National Center of Biotechnology Information.

Prior to RT-qPCR, the various sets of gene-specific primers were tested using a conventional PCR and run in a 2.5% agarose gel stained with ethidium bromide. Only those primers that did not present primer-dimer and a single band at the expected size in the gel were used for RT-qPCR. The accuracy of a primer pairs was also evaluated by the presence of a unique peak during the dissociation step at the end of RT-qPCR. A set of six candidate housekeeping genes was evaluated using geNorm and NormFinder, as described by Vandesompele *et al.*[53] and Andersen *et al.*[54], respectively. The target gene expression levels were calculated using the geometric mean of ribosomal protein S9 (*RSP9)* and succinate dehydrogenase complex subunit A (*SDHA)* as a normaliser.

The efficiency of RT-qPCR amplification for each gene was calculated using the standard curve method with five dilutions at each data point along the curve. Dissociation curves were generated at the end of amplification to verify the presence of a single product.

### Real time quantitative polymerase chain reaction

The RT-qPCR was performed with the StepOne Plus™ Real-Time PCR System, using the Power SYBR® Green master mix (both Applied Biosystems, Foster City, CA, USA). Reaction mixes of 6.25 μL Power SYBR Green master mix (Applied Biosystems, Foster City, CA, USA), 1 μL of forward and reverse primers (160 nM) and 1 μL of diluted cDNA (1:15) template were pipetted into MicroAmp™ optical 96-Well reaction plates and sealed with optical caps (Applied Biosystems, Foster City, CA, USA). After an initial denaturation at 95°C for 10 min, a thermocycling program of 15 s at 95°C, 60 s at 60°C and 15 s at 95°C was applied (40 cycles). Total fluorescence data and dynamic well factors were continuously collected to generate background-subtracted amplification curves (StepOne™ Software version 2.2.2, Applied Biosystems, Foster City, CA, USA). PCR analysis of cDNA samples was performed in duplicate, using no-transcription and no-template samples as controls. The specificity of the PCR amplification was confirmed by melt curve analysis and agarose gel electrophoresis of PCR products.

### Data processing

The PCR efficiency was calculated for each primer set using the StepOnePlus PCR System software (Applied Biosystems), by amplifying 5-fold serial dilutions of pooled cDNA and run in triplicate. The efficiency curves were used to assess accuracy, linearity and efficiency of the PCR reaction. Accuracy was defined as the R^2^ value of the standard curve, efficiency E was calculated as E [%] = (10-1/slope of standard curve - 1) × 100. All primer sets exhibited an efficiency ranged between 85 and 110% and the correlation coefficients were higher than 0.990.

The relative expression (RE) levels were calculated as a variation of the Livak method [55], corrected for variation in amplification efficiency (E = 10^-1/slope^), as shown in Eq. 1.

(1) $$\mathrm{RE}={E_{\mathrm{endogenous}}}^{\left(\mathrm{CT},\mathrm{endogenous}\right)}/{E_{\mathrm{target}}}^{\left(\mathrm{CT},\mathrm{target}\right)}$$

### Statistical analysis

Statistical analyses were carried out with the Statistical Analysis Systems software package, version 9.2, (SAS Institute, Cary, NC, USA). All statistical analyses were performed based on a 2 × 2 factorial arrangement of breed (Alentejana and Barrosã purebreds), diet (high and low silage diets) and their respective interaction. The variances were tested for heteroscedasticity and, for most variables, variance was found to be heterogeneous. Therefore, subsequent data analysis was performed in order to account for heterogeneous variance. The general Satterthwaite approximation was computed in a mixed-effects regression model (PROC MIXED), with breed, diet and their interaction as fixed effects.

The final dataset was analysed using the MIXED procedure of SAS with a model that included breed, diet and their respective interaction as independent variables. Results were expressed as mean ± standard error. Differences between groups were examined for statistical significance using the PDIFF option (Fisher’s test). Differences were significant at *P* < 0.05 and tendencies discussed at *P* < 0.10. Pearson correlation coefficients were calculated using the CORR procedure of SAS.

A principal component analysis (PCA) was performed using the fatty acid composition of liver, intramuscular fat, mesenteric and subcutaneous adipose tissue. The PRINCOMP procedure was applied to a data set of 156 samples and 30 variables to reduce the dimensionality of the data set and to describe the variability of data in two dimensions. After data normalization, the principal components were considered significant if they contributed more than 5% for the total variance.

## Abbreviations

CPT1A: Carnitine palmitoyltransferase 1A; DGAT1: Diacylglycerol O-acyltransferase 1; DHA: Docosahexaenoic acid; ELOVL2: Fatty acid elongase 2; ELOVL5: Fatty acid elongase 5; EPA: Eicosapentaenoic acid; FADS1: Fatty acid desaturase 1; FADS2: Fatty acid desaturase 2; FAME: Fatty acid methyl esters; FASN: Fatty acid synthase; HS: High silage; INSR: Insulin receptor; LC-PUFA: Long chain polyunsaturated fatty acids; LL: *Longissimus lumborum*; LV: Liver; LS: Low silage; NEFA: Non-esterified fatty acids; MAT: Mesenteric adipose tissue; MUFA: Monounsaturated fatty acids; PCA: Principal component analysis; PPARA: Peroxisome proliferator-activated receptor alpha; PUFA: Polyunsaturated fatty acids; RPS9: Ribosomal protein S9; SAT: Subcutaneous adipose tissue; SFA: Saturated fatty acids; SCD: Stearoyl-CoA desaturase; SDHA: Succinate dehydrogenase complex subunit A; SREBF1c: Sterol regulatory element binding transcription factor 1; TFA: Trans fatty acids.

## Competing interests

The authors declare that they have no competing interests.

## Authors’ contributions

ASHC, VMRP and EAR performed the tissue sampling and laboratory work. ASHC was responsible for the statistical analysis. ASHC, RJBB, CMGAF and JAMP were responsible for interpretation of the results and preparation of the manuscript. JAMP and RJBB were responsible for the design of the study. All authors read and approved the findings of the study.
